# Depression and Anxiety Disorders Impact in the Quality of Life of Patients with Inflammatory Bowel Disease

**DOI:** 10.1155/2021/5540786

**Published:** 2021-10-27

**Authors:** Jesús K. Yamamoto-Furusho, Katya E. Bozada Gutiérrez, Andrea Sarmiento-Aguilar, Ana Fresán-Orellana, Perla Arguelles-Castro, Mario García-Alanis

**Affiliations:** ^1^Inflammatory Bowel Disease Clinic, Gastroenterology Department, National Institute of Medical Science and Nutrition Salvador Zubirán, Mexico City, Mexico; ^2^Sub-Direction of Clinical Research, National Institute of Psychiatry Ramón de la Fuente Muñíz, Mexico City, Mexico; ^3^Neurology and Psychiatry Department, National Institute of Medical Science and Nutrition Salvador Zubirán, Mexico City, Mexico

## Abstract

**Objective:**

Anxiety and depression have a negative influence in the quality of life. The aim of the study was to determinate the levels of sensitivity and specificity of the Anxiety and Hospital Depression Scale (HADS) and compare the quality of life in patients with inflammatory bowel disease (IBD) and depression or anxiety.

**Methods:**

This study included 104 patients with diagnosis of IBD. Each patient received psychiatric intervention with SCID-I (Structured Clinical Interview for DSMIV Axis I Disorders) instrument as a gold standard to stablish the cut-off points of HADS. Quality of life was also evaluated with IBDQ-32. Demographic and clinical variables were collected.

**Results:**

Most of the patients reported a high quality of life (73.1%, *n* = 76), while 25.0% (*n* = 26) express a moderate quality of life. The ROC curves for both psychiatric entities showed an adequate discriminative capacity of the HADS-anxiety dimension (AUC = 0.84, 95%CI = 0.76-0.92) with a limited discriminability of the HADS-depression dimension (AUC = 0.58, 95%CI = 0.46-0.70) using the proposed scoring of 8 as a cut-off point.

**Conclusions:**

Anxiety and depression impact negatively in the quality of life in Mexican patients with IBD. The Mexican version of HADS had acceptable internal consistency and external validity, with moderate sensitivity and specificity for clearly identifying clinical cases of anxiety and depression in patients with IBD.

## 1. Introduction

Inflammatory bowel disease (IBD), including Crohn's disease (CD) and ulcerative colitis (UC), is a chronic inflammatory condition that produces damage in the gastrointestinal tract [1] characterized by a relapsing and remitting clinical course where abdominal pain, bloody stools, diarrhea, fever, and weight loss [2] can appear in their mildest form or worsen until the point of compromising life [3]. Epidemiology of IBD varies around the world, but it is clear that it has become an expanding global health problem of industrial-urbanized societies, with a steadily rising incidence and prevalence in developing countries [4]. Recently, the difference between the patients perception of the psychological aspects of IBD and the way physicians identify them and treat them has been recognized [5], and important recommendations have been established emphasizing the importance of identifying psychological problems at routine IBD appointments with the Hospital Anxiety and Depression Scale (HADS), which is considered the most appropriate instrument for this purpose [6]. Together, these actions allow the physician to refer IBD patients when it is appropriate to a mental health specialist [7], considering that ultimately, the final goal of health providers dedicated to the treatment of IBD is to offer a multidisciplinary treatment that achieves clinical, endoscopic, biochemical, and histological remission with a better quality of life (QoL) and avoidance of disability [8].

The relationship among anxiety, depression, and QoL in IBD patients can be influenced by multiple factors; for instance, social isolation, difficult economic state, and maladaptive coping strategies [9], which as has been proposed by Cho et al., can be associated with IBD relapses and the need of surgical treatment [10]. When anxiety and depression are not detected and treated timely, they can become severe, to the point in which generalized anxiety disorder can be considered equivalent to depression in disease burden and reduced psychosocial functioning [11]. Nevertheless, it has yet to be proven what kind of cause-and-effect relationship exists between IBD and anxiety and depression [12], if we consider that these emotional states can aggravate pain and gastrointestinal symptoms by increasing inflammatory activity and finally affecting the QoL [9]. In this same vein, Inflammatory Bowel Disease Questionnaire (IBD-Q32) is a validated and reliable instrument that measures the different emotional, systemic, digestive, and social aspects of IBD that allows us to determine the impact of IBD in the QoL [13]. The IBDQ32 has been already validated in the Mexican IBD population [14], although HADS has already been proven to be a valid instrument in this population [15].

Therefore, the aim of this study was to determine the levels of sensitivity and specificity of the Anxiety and Hospital Depression Scale (HADS) as well as to explore the quality of life in Mexican patients with IBD with psychiatric comorbidity.

## 2. Methods

This is a case control study of IBD patients who attended to their routine follow-up in the IBD clinic at the National Institute of Medical Sciences and Nutrition between the period of August 2017 to February 2018. All the subjects were invited to participate in the study, and a signed informed consent document was provided by all subjects. This study was approved by the local Ethics Committee of our Institution.

## 3. Instruments

### 3.1. Hospital Anxiety and Depression Scale (HADS)

HADS is a self-administered questionnaire that includes 14 interleaved items, 7 of which assess for anxiety symptoms and the other 7 depression symptoms [16]. Anhedonia is the central symptom around which the items of the depression subscale, because it is one of the primary symptoms from which we can distinguish between anxiety and depression [17]. The items are classified in a Likert scale of 4 points (0–3) with a total score that can go from 0 to 21 points in each subscale, where a higher score indicates severity of anxiety or depression symptoms. The questionnaire HADS was given to the patients asking them to answer each question according to the way they have felt during the last 7 days including the day the instrument was applied. The original English version from Zigmond and Snaith in 1983 [18] proposed the following results classification for HADS: 0-7 points absence of anxiety or depression, 8-10 points probable diagnosis, and 11 or more points, suggestive of a clinical problem of anxiety or depression.

### 3.2. Structured Clinical Interview for DSM (SCID-I)

For the external validation of HADS, the Structured Clinical Interview for DSM (SCID-I) was used as the gold standard, which is an interview based on the fourth version of Diagnostic and Statistical Manual of Mental Disorders (DSM-IV). SCID-I is considered an appropriate tool for researchers with a particular interest; it was originally created based on the third version of DSM and posteriorly adapted to the fourth version [19]. To evaluate affective, psychotic, state of mind, substance use, anxiety, somatoform, eating, and adaptive disorders and others, it needs to be done by a trained specialized mental health provider.

### 3.3. Inflammatory Bowel Disease Questionnaire (IBDQ-32)

In order to measure the QoL, an instrument named *Inflammatory Bowel Disease Questionnaire* (IBDQ-32) was used. It was first developed by Guyatt et al. [20] in 1989 and has been widely used in IBD patients, demonstrating to be valid and reliable even in different languages, cultures, and lifestyles including our population [20]. Each item of this questionnaire used a Likert scale of 7 points that provides a total of 32-224 points for the whole questionnaire, classifying in low QoL (32 and 95 points), moderate QoL (96 and 159 points), and high QoL (160 to 224 points). This scale measures 4 dimensions: digestive, systemic, emotional, and social aspects [21]. The affectation is high if the result of points of each question is between the ranges of 10 and 29, average 30 and 49, and low 50 and 70; the systemic symptoms include 5 questions: the affectation is high if the results are between 5 and 14, average of 15 and 24, and low of 25 and 35 points; the emotional symptoms consist of 12 questions in which the affectation is high from 12 to 35, average 36 to 59, and low 60 to 84 points; and in the social with 5 questions, high affectation from 5 to 14, average 25 to 24, and low 25 to 35 points [20].

## 4. Procedure

The total sample of subjects was obtained during the follow-up of the IBD clinic from August 2017 to March 2018. Recruitment was nonprobabilistic using a convenience sampling approach. However, considering an a priori power analysis, assuming equal variances with two-sided research hypotheses, with statistical power of 0.80, alpha value of 0.05 and moderate size effect (0.60) using the G^∗^Power 3.1.9.4 program, a minimal of 90 subjects were required for the present study.

All information was obtained through personal interview and clinical records from all individuals. The inclusion criteria were between age 18 and 60 years old, any gender, and willingness to participate with a previous signed informed consent. All IBD patients with a previous psychiatric disease were excluded of the present study. The demographic and clinical variables analyzed were sex, current age, occupation, patient diagnosis of either CD or UC, age at IBD diagnosis, years of evolution, clinical course, extent or location of disease, extraintestinal manifestations (EIM), and medical treatment.

A specialized psychiatrist applied the SCID-I. The duration of the interview was 1 hour. The data were grouped according to the psychiatric disorder categorized into two main groups: (1) depression disorders (TDM, dysthymic disorder, and substance affect disorder) and (2) anxiety disorders (panic disorder, agoraphobia without panic, social phobia, specific phobia, obsessive-compulsive disorder, posttraumatic stress disorder, generalized anxiety disorder, and substance anxiety disorder).

In order to determine the psychometric properties of HADS, all patients were invited to answer a self-applicable instrument with a total of 14 items which analyzes 2 dimensions (7 items for anxiety and 7 for depression). All points for each dimension were made and established based on the SCID-I interview as the gold standard. The duration to answer the instrument was between 10 and 15 minutes.

For the measurement of the quality of life, the self-applicable specific questionnaire Inflammatory Bowel Disease Questionnaire of 32 items (IBDQ-32) was used, where the information is grouped in 4 dimensions (digestive and systemic, emotional, and social symptoms), having 32 questions that have to answer, selected the option that best suits the way they have felt in the last 2 weeks including the day of the instrument application.

## 5. Statistical Analysis

All demographic and clinical characteristics were evaluated as frequencies and percentages with mean and standard deviations (S.D.). For categorical variables, chi-square tests (*χ*^2^) were used, and for continuous variables, Student's *t*-tests were used. To determine the discriminatory capacity of the HADS scale, a ROC curve (receiver operating characteristic) was plotted and the levels of sensitivity, specificity, false rates, and predictive values were determined for the most used cut-off point of the HADS dimension (8 score). Psychiatric diagnoses obtained through the Structured Clinical Interview for Axis I Disorders of the DSM-IV (SCID-I) was used as the gold standard. In addition, the area under the curve (AUC) was obtained, where values below 0.50 mean a low capacity of the instrument to make an adequate discrimination, while values equal to or greater than 0.80 indicate adequate discrimination. A *p* value < 0.05 was considered significant.

## 6. Results

### 6.1. Demographic and Clinical Characteristics

The sample comprised 104 patients with IBD. A total of 51.9% (*n* = 54) were men and 48.1% (*n* = 50) were women. Their mean age was 41.8 years (S.D. = 11.7, 20-65) with a mean educational level of 12.9 years (S.D. = 4.0, 0-22). At the time of the study, 55.8% (*n* = 58) had a remunerated activity while the remaining 44.2% (*n* = 46) were unemployed.

The majority of the patients had diagnosis of UC (88.5%) and 11.5% with CD. The mean age at diagnosis was 31.2 ± 12.2, and the disease duration was 10.5 ± 7.2 years. Twenty-one percent had at least one extraintestinal manifestation; the presence of arthralgias was the most frequent (50%). All patients were treated with aminosalicylates in 83.7%, steroids 27.9%, thiopurines 29.8% (*n* = 31) and biological treatment such as adalimumab or infliximab 2.9% (*n* = 3) or received surgical treatment (9.6%, *n* = 10) at the time of the study.

Most of the patients reported a high quality of life (73.1%), and 25% had moderate quality of life and only 1.9% showed low quality of life. From the four dimensions assessed in the IBDQ, the dimension of systemic symptoms had a high percentage of moderate and high affectation (34.6%) while less than 30% reported these levels of affectation in the dimensions of bowel symptoms (25.0%), emotional (29.8%), and social functioning (18.3%).

Demographic and clinical features between IBD patients with and without psychiatric comorbidity are shown in Table 1. The significant differences found between both groups were in current age, where patients with depression were older than those without depression and in the IBDQ Emotional Symptoms dimension, where patients with depression exhibited more affection.

### 6.2. Depression and Anxiety in IBD Patients

According to the SCID-I, 24.0% of the patients had a depressive disorder characterized by major depressive disorder (84.0%) and dysthymia (16%). A similar proportion of patients (20.2%) were diagnosed from an anxiety disorder such as generalized anxiety disorder in 76.2% and panic disorder in 38.1%.

Using the already validated HADS Mexican version for patients with IBD, the total score for the depression dimension was of 4.1 ± 3.6 while for the anxiety dimension the total scoring was of 5.4 ± 3.8.

With these scores and the SCID-I, the ROC curves for both psychiatric entities showed an adequate discriminative capacity of the HADS-anxiety dimension (AUC = 0.84, 95% CI: 0.76-.92) with a limited discriminability of the HADS-depression dimension (AUC = 0.58, 95%CI = 0.46-0.70). Nevertheless, adequate sensitivity values were observed in both dimensions using the proposed scoring of 8 as a cut-off point, even though sensibility was low, particularly for the depression dimension.

**Figure 1:**
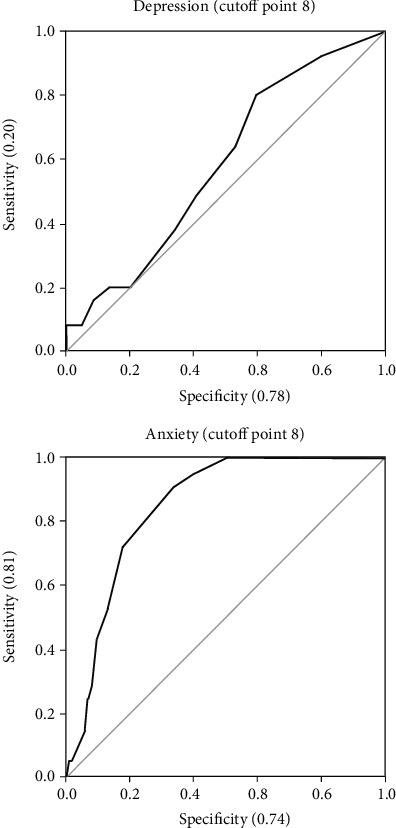
For the depression ROC curve, the following values were determined with the 8 cut-off point: false positive rate (0.22), false negative rate (0.80), positive predictive value (0.23), and negative predictive value (0.76). For the anxiety ROC curve, the following values were determined with the 8 cut-off point: false positive rate (0.17), false negative rate (0.38), positive predictive value (0.48), and negative predictive value (0.90).

The ROC curves, sensibility, specificity, predictive values, and false rates of both dimensions are shown in Figure 1.

In accordance to this, no differences were found between SCID-I and the presence of depressive disorder in the HADS-depression dimension (4.4 ± 3.8 vs. 3.4 ± 3.1, *p* = 0.20). Statistical significant differences were found in the HADS-anxiety dimension between patients with and without SCID-I anxiety disorder (17.1 ± 6.0 vs. 8.4 ± 6.7, *p* < 0.001).

## 7. Discussion

In the present study, we confirmed that the Mexican version of HADS is a reliable and valid tool for screening IBD patients with psychiatric disorders. The questionnaire had good internal consistency and external validity, with favorable sensitivity and specificity similar to previous studies using the original English version of the HADS [22]. It is well known that psychiatric disorders are more prevalent in patients with IBD, and these impact considerably on the quality of life. Previous studies have shown that anxiety and depression are present between 21% and 35%, respectively [23].

We establish the most common cut-off of the HADS dimensions to discriminate patients with anxiety and depression in comparison with the SCID as a gold standard; the scale showed moderate performance for the screening of patients with disorders of anxiety (a cut-off point of 8, AUC for anxiety = 0.84, Sens = 68%, Spec = 83%); however, it was not good for depression disorders (AUC = 0.58, Sens = 20%, Spec = 78%).Although, these results may limit the applicability of the HADS, we should not discard its use as a screening tool for mental disorders such as anxiety disorders or major depression, as it may be a useful clinical tool for daily clinical practice to promote mental health care attention in medically ill patients.

Our results are compared with those obtained in a process of validation and translation into Portuguese, which performed the validation on severe medical pathology (Neurosurgery, Pulmonary, Cardiology, Neurology, and Infectious Illness), in which the best cut-off point to maintain good sensitivity and specificity of the scale was found to be between 4 and 7 points con AUC 0.877 (95% CI: 0.84 to 0.91) similar to our results (cut-off point of 8) [24].

These psychiatric conditions in IBD patients can exacerbate pain and gastrointestinal symptoms by increasing inflammatory activity and impacting in the QoL of the patients by affecting feelings of fatigue and decreasing the motivation needed to overcome the disease [12]. In the present study, we found that 20.2% had anxiety disorder which is lower to that observed in a representative national survey in the French adult population which was estimated to be around 21.6% [25]. It is important to note that significant differences were found in IBD patients with depression older than 60 years versus younger patients 40.7 years (*p* = 0.04). This can be explained because the elderly patients have an increased risk of use of medical care and are often associated with the exposure to multiple drugs, limited mobility, falls, and the cognitive deterioration [26]. Some studies have reported higher rates of psychiatric disorders in IBD compared with other chronic illnesses or healthy controls. Bernstein et al. [27] compared a population-based cohort of IBD cases with community controls from a population-based national health survey; they found a higher prevalence of major depression in IBD cases compared to the community cases (27% vs. 12%).

On the other hand, we found that most of the patients had a high QoL (73.1%) according IBDQ-32, the dimension of systemic symptoms which includes fatigue (34.6%) and emotional symptoms (29.8%) were the most frequent that had moderate to high affection. Our findings are consistent with other studies which have shown that fatigue and emotional symptoms are prevalent in patients with IBD [12, 28, 29].

Czuber-Dochan et al. [30] identified that fatigue prevalence ranges from 22% to 41% when the disease is in remission and around 86% when IBD is active. Fatigue may lead to considerable impairment in the patient's daily life, and three studies have demonstrated that it is associated with reduced quality of life evaluated by IBD-Q32 [31].

The subgroup analysis showed that anxiety disorders had severity in all dimensions of the IBD-Q32 instrument (digestive symptoms, systemic symptoms, emotional symptoms, and social) associated with a low QoL. Therefore, it is clear that anxiety has a negative impact on the life of patients with IBD. A Dutch study of 231 IBD patients reported that up to 43% had high levels of anxiety suggesting that psychiatric complaints in IBD patients are undertreated [32]. The anxiety rate is between 29% and 35% during periods of remission and increases as high as 80% during a relapse of IBD where the correlation between IBD and anxiety levels is strong [11, 33, 34].

In summary, HADS has moderate performance to discriminate anxiety disorders in Mexican IBD patients and the presence of an anxiety disorder is associated with a low QoL. Accurate identification of anxiety and depression, in an early stage in order to provide a specialized treatment for improving the quality of life of IBD patients, is needed. In conclusion, the Mexican version of the HADS had good internal consistency and external validity with favorable sensitivity and specificity for screening of psychiatric disorders in patients with IBD. The patients who had comorbidity and anxiety disorder had lower QoL.

## Figures and Tables

**(a):** 

	Depression present (*n* = 25)			Anxiety present (*n* = 21)		
	*n* (%)	95% C.I.	*p* value	*n* (%)	95% C.I.	*p* value
Gender-female	10 (40.0)	0.6-3.8	0.35	15 (71.4)	0.1-0.8	0.01
Occupation-employed	17 (68.0)	0.1-1.3	0.15	9 (42.9)	0.7-5.0	0.18
Ulcerative colitis	23 (92.0)	0.1-2.9	0.52	19 (90.5)	0.1-3.8	0.74
EIMs- present	3 (12.0)	0.1-1.5	0.19	5 (23.8)	0.3-3.7	0.73
Quality of life^+^						
Digestive symptoms	9 (36.0)	0.7-5.4	0.14	12 (57.1)	2.3-18-5	<0.001
Systemic symptoms	10 (40.0)	0.5-3.4	0.51	15 (71.4)	2.5-21.4	<0.001
Emotional symptoms	10 (40.0)	0.7-4.7	0.20	13 (61.9)	2.1-16.3	<0.001
Social	6 (24.0)	0.5-4.7	0.39	9 (42.9)	1.8-16.2	<0.001
Total	9 (36.0)	0.6-4.6	0.24	13 (61.9)	2.5-20.9	<0.001

**(b):** 

	Depression		*p* value	Anxiety		*p* value
	Present	Absent		Present	Absent	
	Mean (S.D)			Mean (S.D)		
Age (years)	45.2 (8.2)	40.7 (12.5)	0.04	43.2 (12.2)	41.4 (11.6)	0.52
Level of education (years)	12.9 (3.1)	12.9 (4.2)	0.99	12.4 (3.9)	13.0 (4.0)	0.57
Age of diagnosis (years)	31.7 (10.5)	31.0 (12.7)	0.81	33.6 14.3	30.6 (11.6)	0.31
Illness evolution (years)	13.5 (9.2)	9.6 (6.3)	0.06	9.6 9.3	10.8 (6.6)	0.51

IBD: inflammatory bowel disease; EIMs: extraintestinal manifestations. ^+^The percentages reported are for patients with moderate to severe affections in IBDDQ-32 dimensions. The percentages reported are for patients with moderate to severe affections in IBDQ-32 dimensions.

## Data Availability

Data availability will be released upon request.
